# Bone Resorption After Microvascular Osseous Free-Flap Reconstruction in Pediatric Patients: A Retrospective Study

**DOI:** 10.7759/cureus.116159

**Published:** 2026-09-12

**Authors:** Maciej Borowiec, Jeremi Matysek, Dominika Lech, Robert Maksymowicz, Cyprian Straczek, Marcin Kozakiewicz, Lukasz Krakowczyk, Krzysztof Dowgierd

**Affiliations:** 1 Department of Maxillofacial, Reconstructive and Plastic Surgery, Head and Neck Surgery Clinic for Children and Young Adults, Regional Specialized Children’s Hospital in Olsztyn, Olsztyn, POL; 2 Department of Clinical Pediatrics, Head and Neck Surgery Clinic for Children and Young Adults, University of Warmia and Mazury in Olsztyn, Olsztyn, POL; 3 Department of Maxillofacial Surgery, Medical University of Lodz, Lodz, POL; 4 2nd Department of Oncologic Surgery, Maria Skłodowska-Curie National Research Institute of Oncology, Gliwice, POL

**Keywords:** bone resorption, dental rehabilitation, fibula free flap, free tissue flaps, head and neck reconstruction, microvascular reconstruction, pediatric patients

## Abstract

Background

Bone resorption is an important consideration following microvascular osseous reconstruction. However, data regarding clinically significant bone resorption in pediatric patients remain limited. This study aimed to assess the occurrence of clinically significant bone resorption six months after reconstruction with vascularized bone-containing free flaps in pediatric patients and to evaluate whether the occurrence of major bone resorption differed according to flap type.

Methods

This retrospective observational study included 46 pediatric patients who underwent 54 microsurgical free-flap procedures between 2013 and 2017. After exclusion of five soft-tissue flaps and 13 necrotic bone-containing flaps, 36 viable osseous free flaps were included in the bone-resorption analysis. Clinically significant bone resorption was assessed on computed tomography performed six months after reconstruction using a modified radiographic bone-height assessment based on the adjacent dentition. Major bone resorption was defined as insufficient reconstructed bone height to accommodate a dental implant of a length typically used for the corresponding anatomical region. The occurrence of major bone resorption at six months was compared according to free-flap type using Fisher’s exact test.

Results

Major bone resorption was observed in 8 of 36 evaluable flaps (22.2%), whereas 28 flaps (77.8%) showed no major resorption. Major resorption occurred in 6 of 25 fibular flaps (24.0%) and two of nine iliac crest flaps (22.2%). No major resorption was observed among the two medial femoral condyle flaps. No statistically significant association was found between flap type and major bone resorption (Fisher’s exact test, p = 1.000).

Conclusion

Clinically significant bone resorption may occur during the early postoperative period following microvascular osseous reconstruction in pediatric patients. Postoperative radiological assessment of reconstructed bone may be particularly valuable when future dental rehabilitation is anticipated.

## Introduction

Bone resorption is an important factor influencing the long-term outcomes of reconstructive procedures involving vascularized osseous free flaps. Although postoperative remodeling of transplanted bone is an expected biological process, excessive loss of bone height may compromise the structural and functional objectives of reconstruction. This is particularly relevant in the maxillofacial region, where reconstructed bone may subsequently be used for dental rehabilitation and implant placement. Preservation of adequate bone dimensions is therefore an important component of successful long-term reconstruction.

Several studies have investigated postoperative bone changes following vascularized osseous free-flap reconstruction, particularly in the mandible. Progressive atrophy of vascularized fibular grafts and longitudinal changes in bone height have been reported following mandibular reconstruction [1,2]. Three-dimensional computed tomography (CT) studies have demonstrated differences in postoperative volume changes among different types of vascularized bone flaps [3]. More recent studies have further characterized postoperative bone changes using CT-based measurements of fibular and iliac crest flaps [4,5].

However, most available evidence concerning postoperative bone resorption following microvascular reconstruction is derived from adult populations. Pediatric patients represent a distinct population because reconstruction is performed in the setting of ongoing craniofacial growth and skeletal remodeling. Previous studies of pediatric mandibular reconstruction have primarily focused on mandibular growth and long-term functional outcomes, while data specifically addressing clinically relevant bone resorption remain limited [6-10].

In addition, previous studies have predominantly quantified postoperative bone loss using linear measurements or three-dimensional volumetric analysis [1,3,9-12]. From a clinical perspective, however, preservation of sufficient bone height for subsequent dental implant placement may represent a more directly relevant outcome. Adequate vertical bone dimensions are particularly important in the maxillofacial region when secondary dental rehabilitation is anticipated. Therefore, assessment based on the clinical adequacy of reconstructed bone may complement quantitative measurements of bone volume and provide information more directly relevant to subsequent treatment.

The present study aimed to assess the occurrence of clinically significant bone resorption six months after reconstruction with vascularized bone-containing free flaps in pediatric patients. The secondary aim was to evaluate the association between free-flap type and the occurrence of clinically significant bone resorption at six months.

## Materials and methods

Study design and population

This retrospective observational study included pediatric patients with head and neck tumors, congenital craniofacial defects, or acquired craniofacial defects who underwent reconstruction using microsurgical free flaps at the Department of Head and Neck Surgery with the Maxillofacial Surgery Unit of the Regional Specialist Children’s Hospital in Olsztyn, Poland, between 2013 and 2017. Inclusion criteria were pediatric age (16 months to 18 years), reconstruction of a head and neck defect using a microsurgical free flap, and availability of postoperative clinical and radiological records for assessment. For the bone-resorption analysis, only viable free flaps containing an osseous component were included. Soft-tissue-only flaps and bone-containing flaps that developed flap necrosis were excluded from the bone-resorption analysis.

The study was initiated in 2017 following approval by the Bioethics Committee on September 29, 2017 (approval no. KB/430-15/17). The study was conducted in accordance with the principles of the Declaration of Helsinki. Informed consent for treatment and open-access publication was obtained or waived for all participants, as applicable. The clinical data were collected between 2013 and 2017, whereas the available clinical and radiological records were comprehensively verified and analyzed during the subsequent continuation of the project and preparation of the present manuscript. The study population comprised 46 patients, including 22 boys and 24 girls, aged 16 months to 18 years, who underwent a total of 54 microsurgical procedures. Twenty-six patients underwent tumor resection with immediate reconstruction, whereas 20 underwent reconstructive procedures without preceding tumor resection. The latter group included 18 patients with craniofacial deformities, one patient with a chemical burn of the oral cavity, and one patient with a post-traumatic maxillary bone defect. The manuscript was reviewed and revised in accordance with the Strengthening the Reporting of Observational Studies in Epidemiology (STROBE) recommendations for observational studies.

Free flap selection and study population for bone-resorption analysis

Five types of free flaps were used in the study population: fibular, iliac crest, radial forearm, anterolateral thigh, and medial femoral condyle flaps. Of the 54 microsurgical free-flap procedures, five soft-tissue flaps were excluded from the bone-resorption analysis, leaving 49 bone-containing flaps for further assessment. Thirteen flaps were subsequently excluded because of flap necrosis. Some patients underwent more than one microsurgical procedure because of reoperation following failure and necrosis of a previous free flap. These repeat procedures did not represent additional eligible osseous flaps from the same patient for the final bone-resorption analysis, as the previously failed flaps were excluded because of necrosis. The final analysis therefore included 36 viable bone-containing flaps, each derived from a different patient. These comprised 25 fibular flaps, nine iliac crest flaps, and two medial femoral condyle flaps.

Assessment of bone resorption

Clinically significant postoperative bone resorption was assessed on CT images obtained six months after microvascular reconstruction. All flaps included in the bone-resorption analysis were evaluated at this postoperative time point. The assessment focused on the postoperative height of the osseous component of the free flap rather than on volumetric bone loss.

CT images were analyzed in the coronal plane, selected to visualize the long axis of the tooth located closest to the osteotomy line. In dentate segments of the maxilla and mandible, reconstructed bone height was assessed using a modified radiographic approach based on the method described by Abyholm et al. [13], in which bone height is evaluated in relation to the adjacent dentition. For the purposes of the present study, this approach was adapted to assess the clinical adequacy of the reconstructed bone for potential subsequent dental implant rehabilitation.

At the six-month postoperative assessment, the reconstructed bone was classified as showing no major bone resorption when its vertical height at the recipient site was sufficient to accommodate a dental implant of a length typically used for the corresponding anatomical region of the maxilla or mandible in a patient without previous free-flap osseous reconstruction. Major bone resorption was defined as insufficient residual bone height to accommodate such an implant, as illustrated in Figure 1. Accordingly, each viable bone-containing free flap was classified at six months after reconstruction into one of two categories: no major bone resorption or major bone resorption. The radiological assessment was performed jointly by a radiologist and a maxillofacial surgeon.

**Figure 1:**
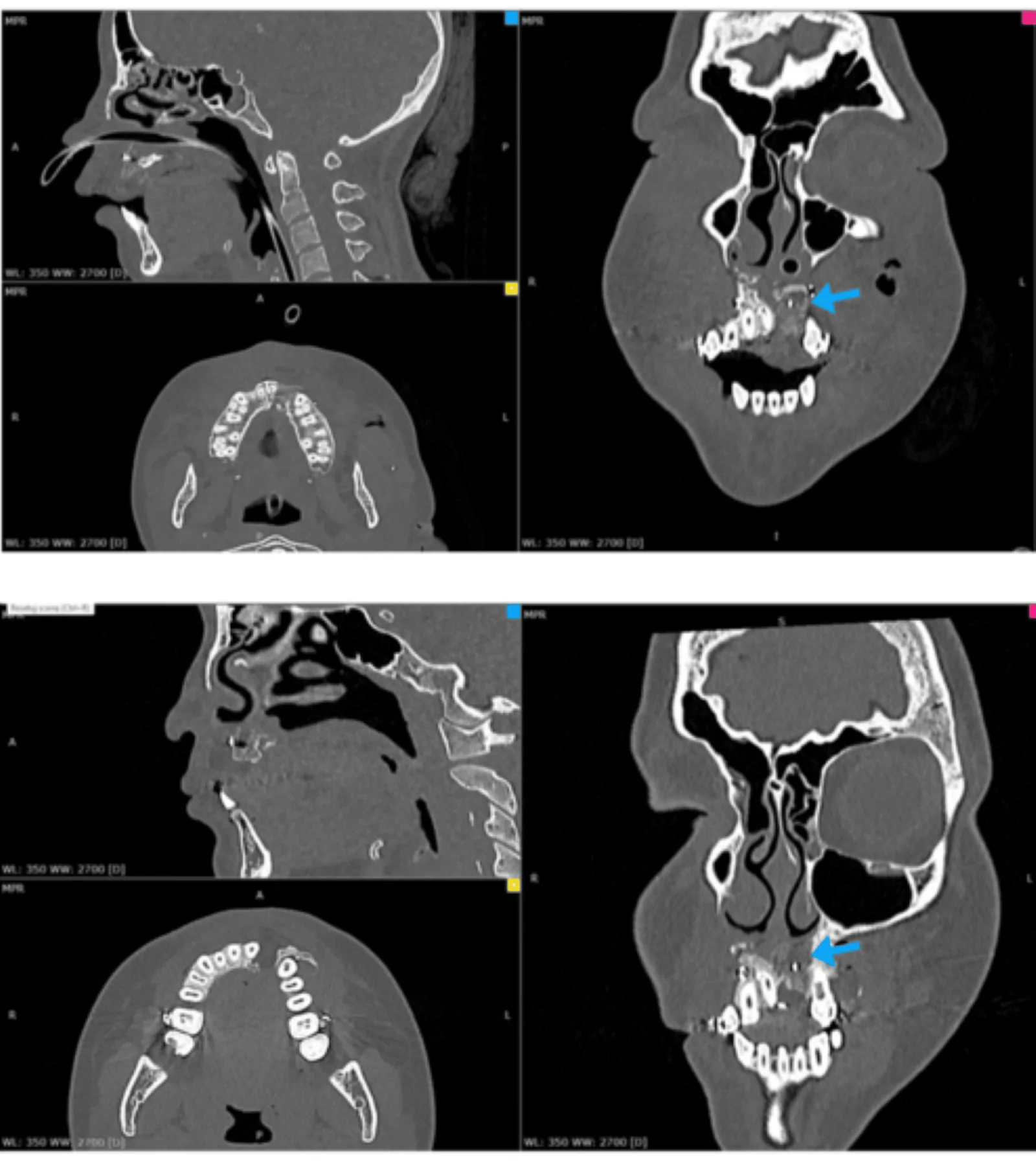
Postoperative computed tomography demonstrating major bone resorption of a vascularized iliac crest free flap used for reconstruction of an alveolar cleft defect in the region of tooth 22 The arrow indicates the transplanted osseous flap with insufficient residual bone height for subsequent dental implant placement.

Statistical analysis

Statistical analyses were performed using STATISTICA 13.3 PL (StatSoft, Kraków, Poland). The association between the type of bone flap and the occurrence of major bone resorption at six months after reconstruction was assessed using Fisher’s exact test. The analysis included the 36 viable bone-containing flaps that were evaluable for bone resorption. Statistical significance was set at α = 0.05.

## Results

A total of 54 microsurgical free-flap procedures were performed in 46 pediatric patients. The demographic and clinical characteristics of the study population are summarized in Table 1. Five soft-tissue-only flaps were excluded from the bone-resorption analysis. Of the remaining 49 bone-containing flaps, 13 were excluded because of flap necrosis. Therefore, 36 viable bone-containing free flaps were available for the final analysis of postoperative bone resorption, as shown in Figure 2. Each of the 36 viable osseous free flaps included in the final analysis was derived from a different patient. Of these 36 viable bone-containing free flaps, 14 (38.9%) were used for maxillary reconstruction and 22 (61.1%) for mandibular reconstruction.

**Table 1:** Demographic and clinical characteristics of the study population

Variable	n (%)
Patients	46
Age range	16 months-18 years
Sex	
Male	22 (47.8)
Female	24 (52.2)
Indication for reconstruction	
Tumor resection with immediate reconstruction	26 (56.5)
Reconstruction without preceding tumor resection	20 (43.5)
Craniofacial deformity	18 (39.1)
Chemical burn of the oral cavity	1 (2.2)
Post-traumatic maxillary bone defect	1 (2.2)
Site of reconstruction among viable osseous flaps (n = 36)	
Maxilla	14 (38.9)
Mandible	22 (61.1)

**Figure 2:**
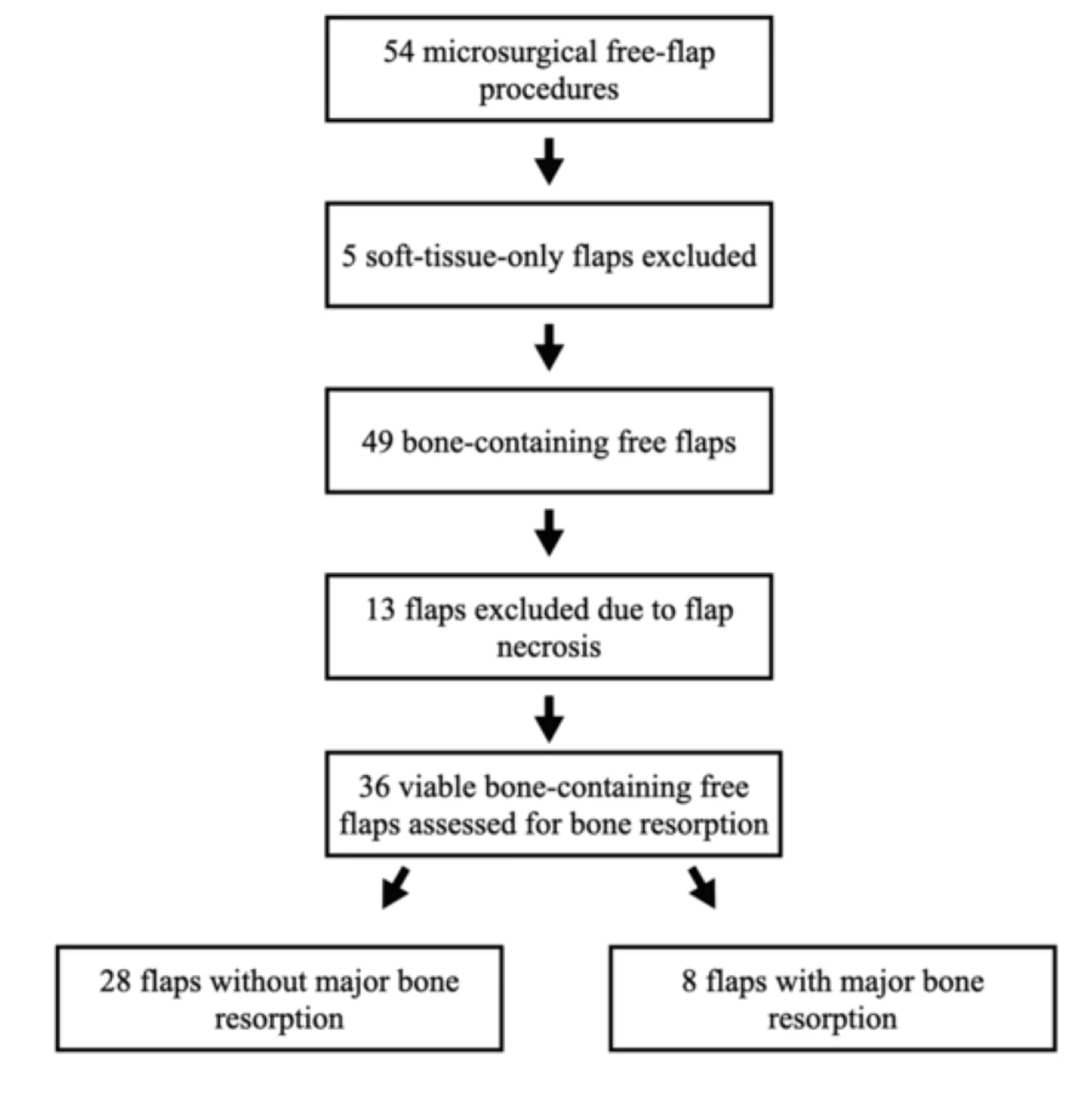
Flowchart of patient and free-flap selection for the final analysis of postoperative bone resorption

Among the 54 microsurgical free-flap procedures, 29 were fibular free flaps, 14 were iliac crest free flaps, six were radial forearm flaps, three were anterolateral thigh flaps, and two were medial femoral condyle flaps, as shown in Table 2.

**Table 2:** Characteristics of free flaps used in the study

Flap type	Total, n (%)	Osseous, n	Soft-tissue, n
Fibular free flap	29 (53.7%)	29	0
Iliac crest free flap	14 (25.9%)	14	0
Radial forearm flap	6 (11.1%)	4	2
Anterolateral thigh (ALT) flap	3 (5.6%)	0	3
Medial femoral condyle flap	2 (3.7%)	2	0
Total	54 (100%)	49	5

Among the 36 viable bone-containing flaps included in the final analysis, 25 were fibular flaps, nine were iliac crest flaps, and two were medial femoral condyle flaps. Major bone resorption was observed in eight of 36 flaps, representing 22.2% of the analyzed flaps, whereas 28 flaps, representing 77.8%, showed no major bone resorption. Of the eight flaps with major bone resorption, six were fibular flaps and two were iliac crest flaps. No cases of major bone resorption were observed among the two medial femoral condyle flaps, as shown in Table 3.

**Table 3:** Major bone resorption according to flap type

Flap type	No major resorption, n (%)	Major resorption, n (%)	Total, n
Fibular	19 (76.0%)	6 (24.0%)	25
Iliac crest	7 (77.8%)	2 (22.2%)	9
Medial femoral condyle	2 (100%)	0 (0%)	2
Total	28 (77.8%)	8 (22.2%)	36

Fisher’s exact test showed no statistically significant association between flap type and the occurrence of major bone resorption at six months (p = 1.000).

## Discussion

In the present study, major bone resorption was identified in 8 of 36 viable bone-containing free flaps evaluated six months after reconstruction, corresponding to 22.2% of the analyzed flaps. This finding indicates that clinically relevant loss of reconstructed bone height may already be detectable six months after microvascular osseous reconstruction in pediatric patients. Importantly, the endpoint used in this study was based on the clinical adequacy of the reconstructed bone for potential subsequent dental implant placement rather than on a percentage reduction in bone volume. This distinction is important when interpreting our findings in relation to previous studies, which have assessed postoperative bone changes using linear measurements, such as bone height, as well as three-dimensional volumetric or CT-based analyses [1,3,5,9-12]. The clinically oriented endpoint used in the present study was intended to identify bone loss that could have direct implications for subsequent dental rehabilitation. However, it does not quantify the absolute or relative amount of bone lost. Consequently, this approach may underestimate the extent of bone resorption when measurable bone loss has occurred, but the remaining bone height is still sufficient for potential implant placement. Conversely, classification as major bone resorption does not necessarily indicate a large percentage reduction in total bone volume, as a relatively limited reduction in vertical bone height may be sufficient to make the reconstructed site inadequate for implant placement. Therefore, the present endpoint should be interpreted as a clinically relevant threshold rather than a quantitative measure of the total extent of postoperative bone resorption. Differences in assessment methods should be taken into account when comparing our findings with studies based on linear or volumetric measurements.

Previous studies have demonstrated that postoperative remodeling and resorption of vascularized bone may continue over an extended period following reconstruction. Progressive atrophy of vascularized fibular grafts and longitudinal changes in bone dimensions have been reported following mandibular reconstruction [1,2]. Three-dimensional CT studies have demonstrated differences in bone volume loss among fibular, iliac crest, and scapular flaps, with the greatest loss reported for scapular flaps [3]. Similarly, postoperative cortical bone resorption has been described following maxillary reconstruction with a vascularized fibular flap, while reductions in the dimensions and density of vascularized iliac bone have also been reported following maxillary reconstruction [4,5]. Together, these findings indicate that postoperative bone remodeling and resorption have been consistently reported across different types of vascularized osseous free flaps and reconstructive sites.

The pediatric population requires particular consideration because reconstruction takes place in the setting of ongoing craniofacial growth and skeletal remodeling. Evidence regarding the long-term behavior of vascularized bone flaps in children remains more limited than that available for adults. Pediatric mandibular reconstruction studies have emphasized the importance of residual mandibular growth, condylar preservation, and the timing of reconstruction in determining subsequent mandibular development [6]. Long-term clinical observations have demonstrated satisfactory functional and aesthetic outcomes following fibular flap reconstruction in children [8]. The continued development of the reconstructed mandible may reflect growth of the residual native mandible rather than intrinsic growth of the transplanted fibula [7,10]. Serial three-dimensional CT studies have demonstrated postoperative changes involving both the grafted fibula and the residual mandible, while the height of the grafted fibula may remain relatively stable during long-term follow-up [7]. These findings support the concept that postoperative changes in reconstructed bone in children reflect the interaction of resorption, remodeling, and ongoing craniofacial growth rather than a simple, linear process of bone loss.

In our cohort, major bone resorption occurred in 6 of 25 fibular flaps (24.0%) and in two of nine iliac crest flaps (22.2%), while no cases were observed among the two medial femoral condyle flaps. No statistically significant association was found between flap type and major bone resorption (Fisher’s exact test, p = 1.000). However, these findings should be interpreted cautiously because of the small number of patients within individual flap groups, particularly the medial femoral condyle group. The absence of a statistically significant association should therefore not be interpreted as evidence that flap type has no influence on postoperative bone remodeling. Previous studies have suggested that postoperative bone behavior may differ according to flap type [3]. Changes in bone mineral density and cortical bone thickness have been reported following fibular reconstruction, with preservation of the native mandibular condyle identified as a potential factor associated with postoperative bone changes [14].

Three-dimensional CT analysis has demonstrated volume reduction in vascularized scapular and fibular grafts and an association between bone resorption and follow-up time [9]. Differences between these findings and the present results may reflect differences in patient age, anatomical site, indication for reconstruction, follow-up duration, and methods used to assess bone changes.

The clinical relevance of bone preservation extends beyond maintenance of reconstructed skeletal volume. In the maxillofacial region, adequate vertical bone dimensions may be important for subsequent dental rehabilitation and implant placement. Previous pediatric series have demonstrated that dental rehabilitation is an important component of long-term management following microvascular maxillofacial reconstruction [8,15]. A systematic review of pediatric maxillofacial reconstruction with vascularized fibular flaps reported that 43 of 155 patients (27.7%) underwent or awaited dental rehabilitation [15]. Consequently, assessment based on the adequacy of reconstructed bone for future implant placement may provide clinically relevant information that complements quantitative measurements of bone volume. Preservation of adequate vertical bone height may be particularly relevant when secondary implant-supported dental rehabilitation is anticipated.

Bone remodeling following microvascular reconstruction is a dynamic process influenced by biomechanical and anatomical factors and by interactions between transferred bone and the native skeleton. Clinical and biomechanical studies have demonstrated time-dependent changes in bone remodeling following mandibular reconstruction with vascularized fibular flaps [16]. In pediatric patients, these processes may be further influenced by ongoing craniofacial growth and changes in the reconstructed mandibular complex [6,7]. Therefore, the finding of clinically significant bone resorption at six months should be interpreted as an early postoperative finding within a broader and dynamic remodeling process rather than as evidence of the final long-term dimensions of the reconstructed bone.

Limitations

Several limitations should be considered when interpreting the present findings. First, the retrospective design and relatively small sample size limit statistical power, particularly for comparisons between individual flap types. Second, the number of medial femoral condyle flaps was too small to permit meaningful comparison with fibular and iliac crest flaps. Third, the six-month follow-up represents a relatively short observation period and does not allow assessment of longer-term bone remodeling. Bone resorption was assessed using a clinically oriented radiological criterion rather than standardized quantitative measurements of bone height or three-dimensional bone volume. The assessment did not include three-dimensional angular measurements of reconstructed bone. The classification was based on the joint evaluation of reconstructed bone height and its potential adequacy for subsequent dental implant placement, without a predefined numerical threshold for bone height. Although this approach reflects the potential clinical relevance of reconstructed bone dimensions for subsequent implant rehabilitation, it does not provide a quantitative estimate of the extent of bone loss and may be subject to misclassification. In addition, the cohort included patients with different underlying conditions, reconstructive indications, and anatomical sites, all of which may influence postoperative bone remodeling. Finally, only viable bone-containing flaps that were evaluable at six months were included in the final analysis, which may have introduced selection bias.

Despite these limitations, the present study provides clinically relevant information on the occurrence of major bone resorption following microvascular osseous reconstruction in pediatric patients. Postoperative radiological assessment may be particularly relevant when secondary dental rehabilitation is anticipated. Larger prospective studies incorporating standardized quantitative three-dimensional imaging, longer follow-up, and clinical dental rehabilitation outcomes are warranted to further characterize the incidence, time course, and determinants of postoperative bone remodeling in pediatric patients.

## Conclusions

Clinically significant bone resorption may occur during the early postoperative period following microvascular osseous reconstruction in pediatric patients. Postoperative radiological assessment of reconstructed bone may be particularly valuable when future dental rehabilitation is anticipated.

Further prospective studies with longer follow-up and standardized quantitative imaging are needed to better characterize postoperative bone remodeling and identify factors associated with clinically relevant bone resorption.
